# Supplementary figures and images for: Modeling the impact of xenointoxication in dogs to halt Trypanosoma cruzi transmission
Source: PLoS Comput Biol. 2023 May 8;19(5):e1011115. doi: 10.1371/journal.pcbi.1011115 (PMC10194993; doi:10.1371/journal.pcbi.1011115)

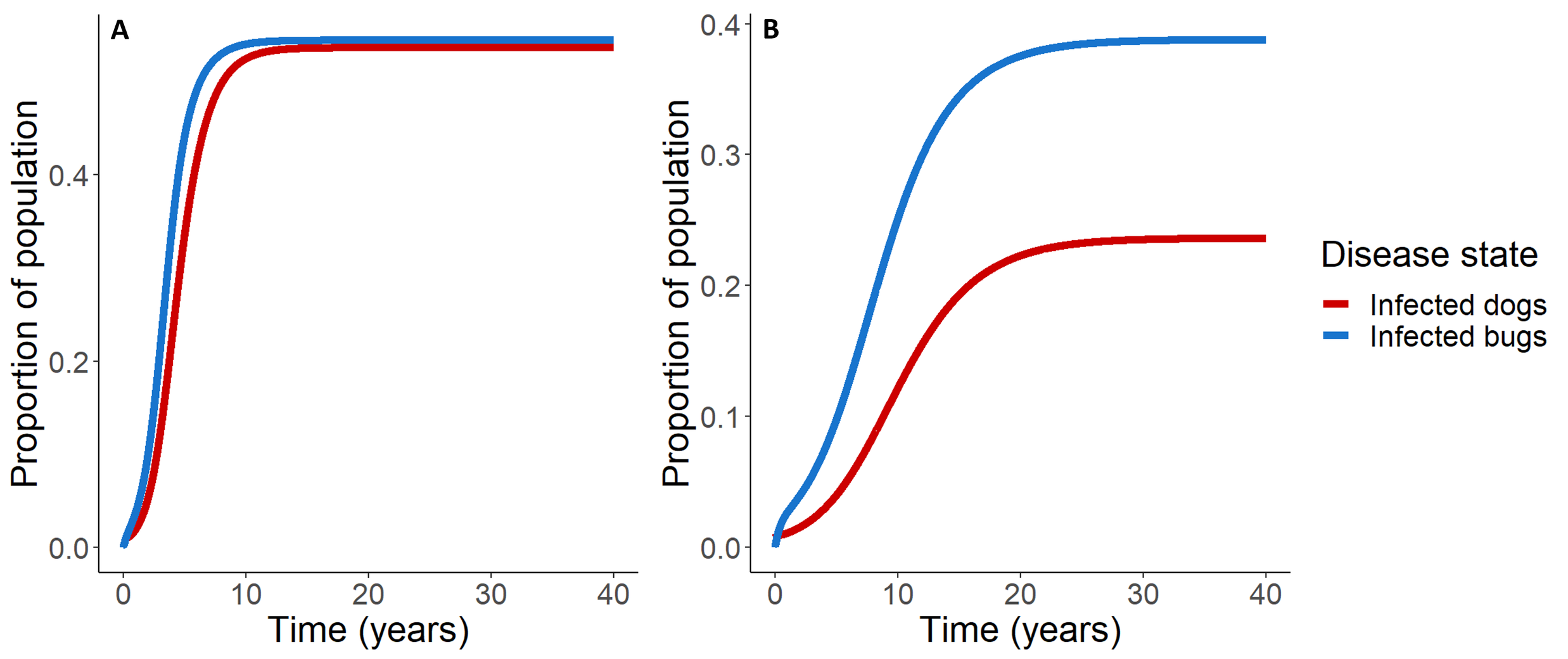

Supplement: S1 Fig — A corresponds to the baseline pre-treatment model in regions with high disease prevalence and domestic vectors. B corresponds to the baseline pre-treatment model in regions with low prevalence and sylvatic vectors. (TIF) [file pcbi.1011115.s001.tif]

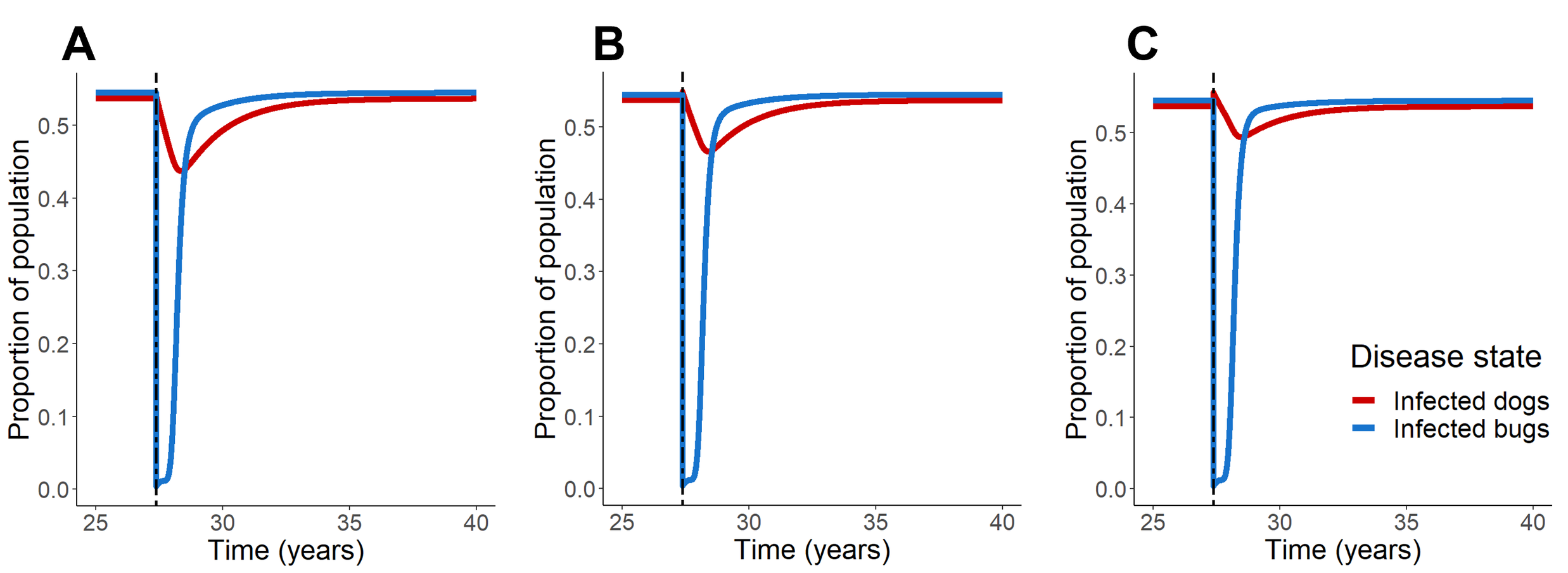

Supplement: S2 Fig — (A) corresponds to 20% of bugs being consumed; (B) corresponds to 50% of bugs being consumed; (C) corresponds to 80% of bugs being consumed. (TIF) [file pcbi.1011115.s002.tif]

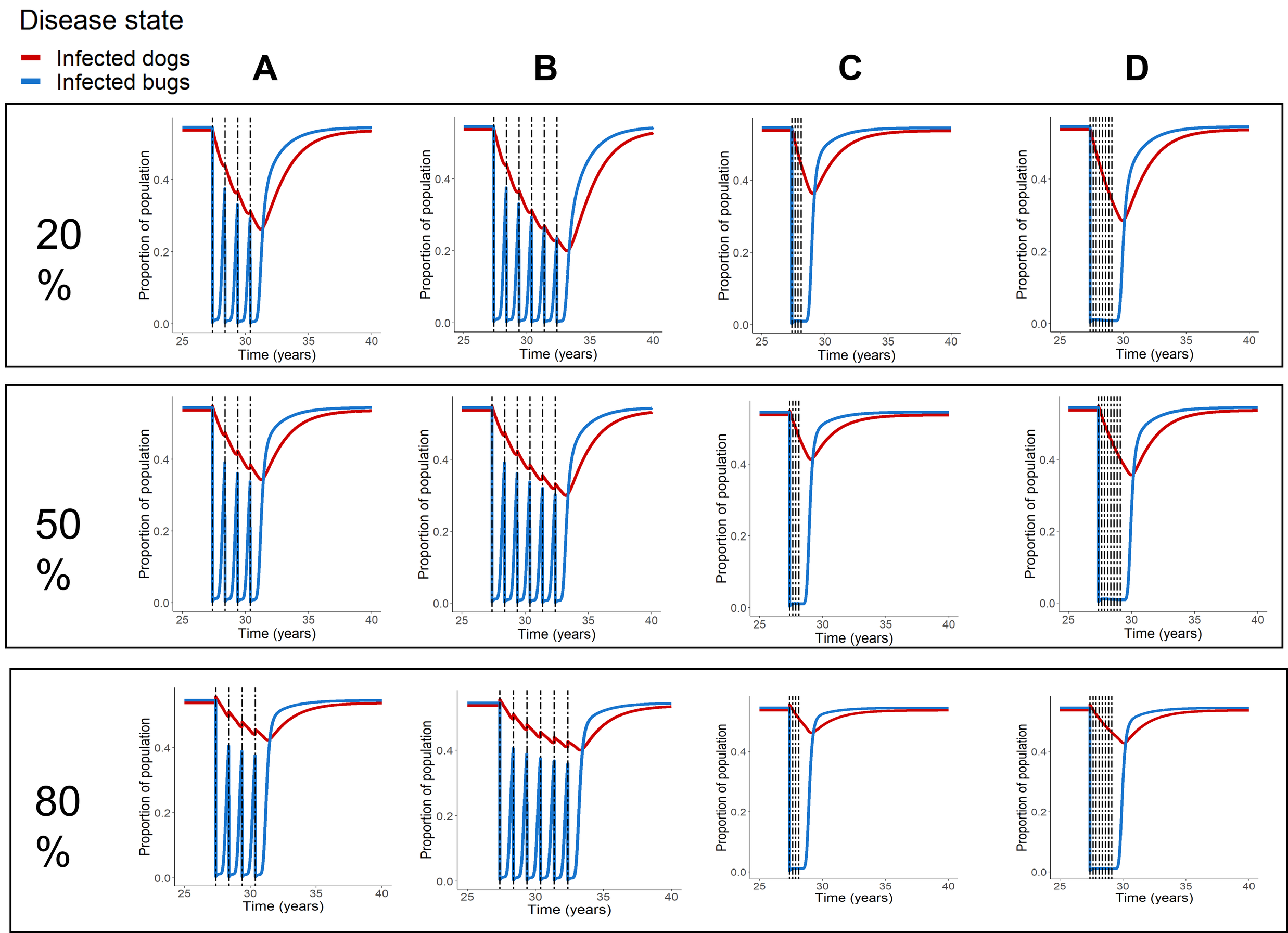

Supplement: S3 Fig — Annual administration of fluralaner for both 4 years (A) and 6 years (B) was simulated, as well as administration every 90 days (veterinary recommendation) for one year (C) and for two years (D). (TIF) [file pcbi.1011115.s003.tif]

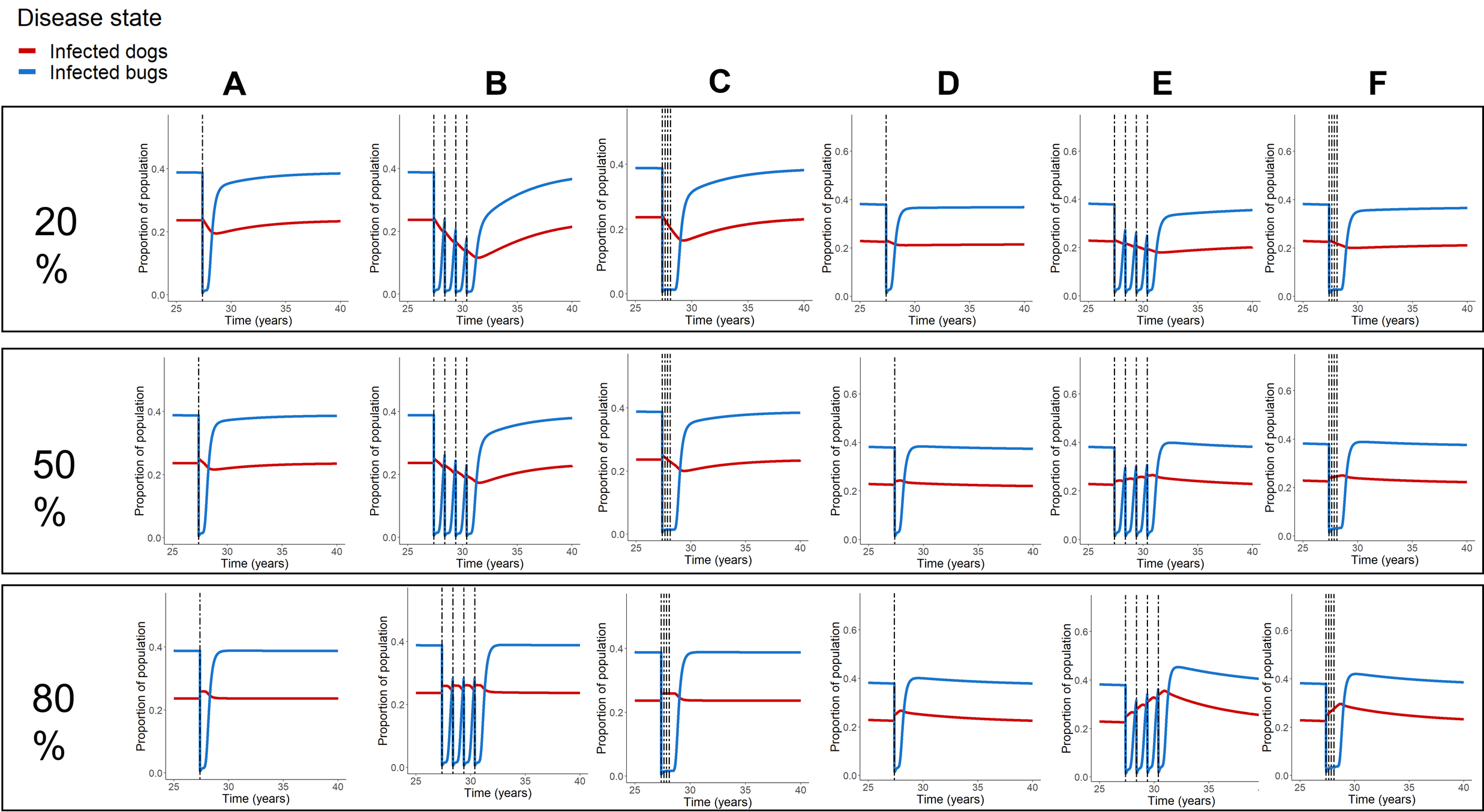

Supplement: S4 Fig — We explored a range of dog average lifespan from 3 years (A-C) to 6 years (D-F). Treatment scenarios include one time treatment (A, D), annual treatment for 4 years (B, E), and treatment every 90 days for 1 year (C, F). (TIF) [file pcbi.1011115.s004.tif]

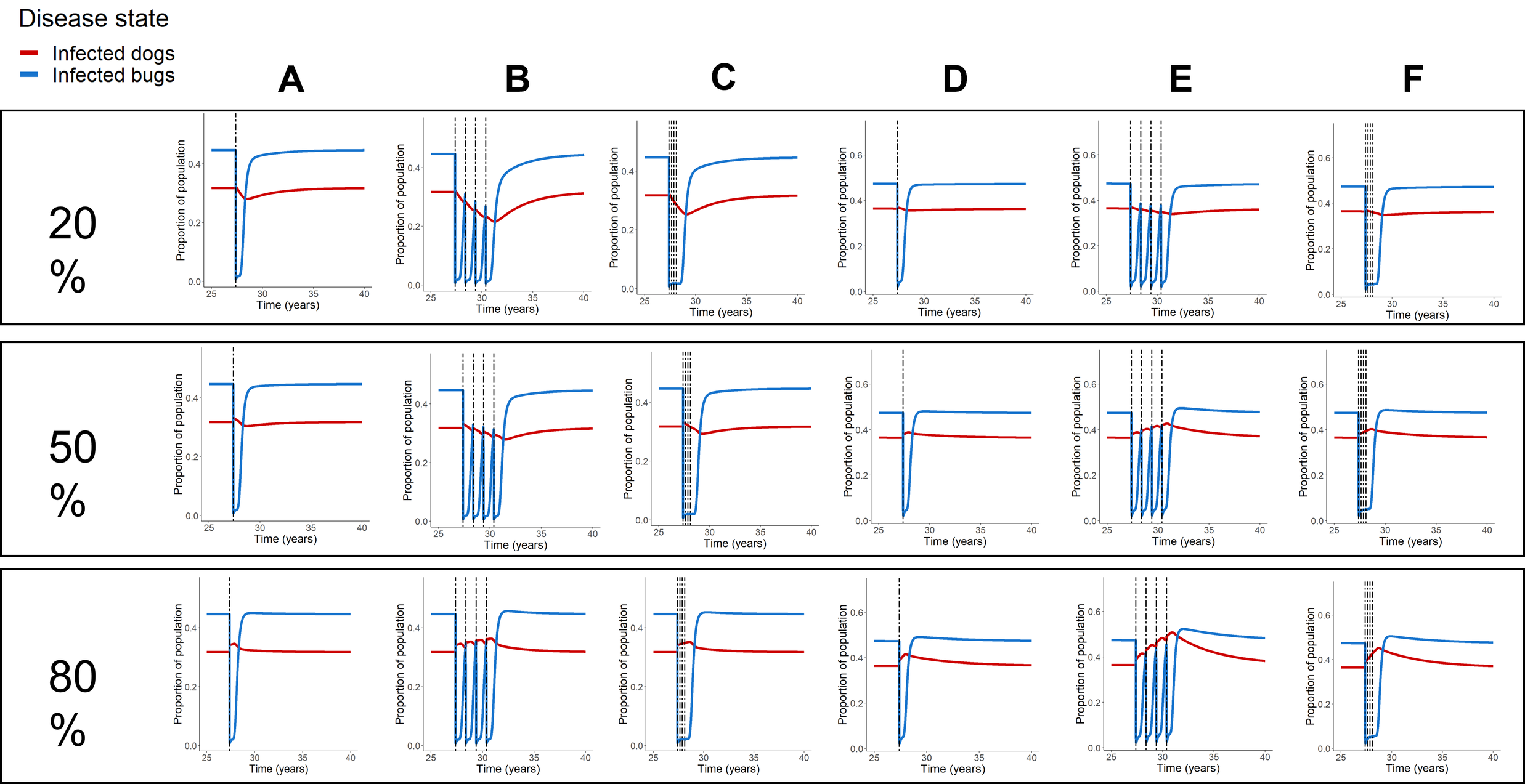

Supplement: S5 Fig — We explored a range of dog average lifespan from 3 years (A-C) to 6 years (D-F). Treatment scenarios include one time treatment (A, D), annual treatment for 4 years (B, E), and treatment every 90 days for 1 year (C, F). (TIF) [file pcbi.1011115.s005.tif]

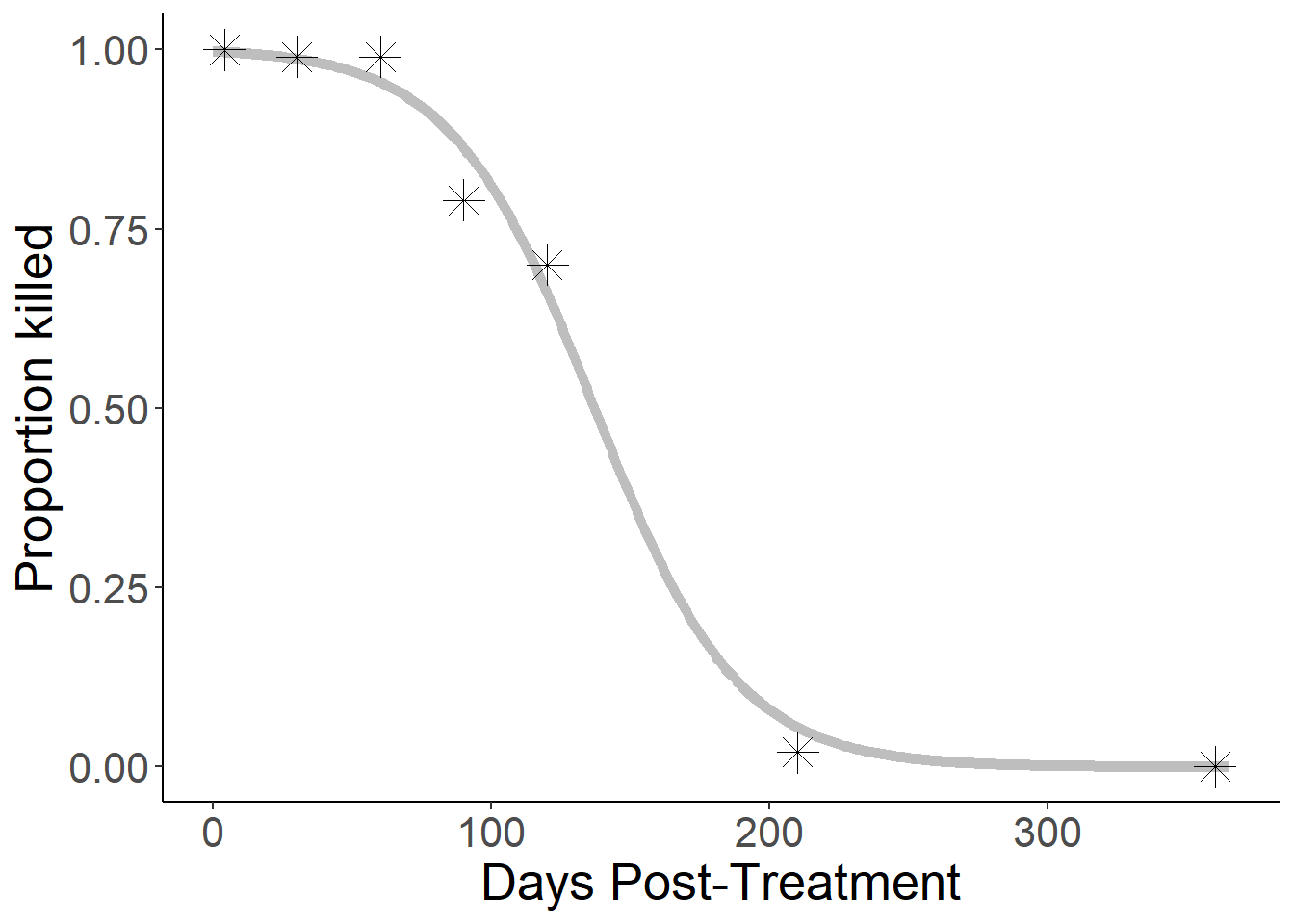

Supplement: S6 Fig — Data from Laino et al (2019) on the declining percentage of bugs killed after feeding on fluralaner treated dogs was fit to a logistic curve and incorporated into the model of T. cruzi transmission dynamics in bugs and dogs with fluralaner treatment. Initial analyses used the data from 5th stage pyrethroid susceptible nymphs. (TIF) [file pcbi.1011115.s006.tif]
