# Supplementary material for: Modeling the impact of xenointoxication in dogs to halt Trypanosoma cruzi transmission
Source: PLoS Comput Biol. 2023 May 8;19(5):e1011115. doi: 10.1371/journal.pcbi.1011115 (PMC10194993; doi:10.1371/journal.pcbi.1011115)
Supplement: Attachment — Submitted filename: PLOS One Paper review.docx [file pcbi.1011115.s008.docx]

**PLOS One Paper review**

Thank you for the opportunity to review this paper. I found this paper interesting and well-written. However, I have the following minor comments/questions/suggestions.

Page 88: Oral transmission through predation of infected vectors is thought the be the most frequent mechanism of infection among hosts in the sylvatic cycle. Looks like a grammatical error in this sentence.

Line 127-128: What are the potential pathological reasons that make the killed bugs still infectious? Please elaborate with citations.

Line 141: typo “.” After 38.81%

Line 452-453

What are the possible reasons that make dogs consume dead bugs? Are there any behavioral

Table 1: What are the units for the parameter values (such as incubation period) in table 1.
