# Supplementary material for: Modeling the impact of xenointoxication in dogs to halt Trypanosoma cruzi transmission
Source: PLoS Comput Biol. 2023 May 8;19(5):e1011115. doi: 10.1371/journal.pcbi.1011115 (PMC10194993; doi:10.1371/journal.pcbi.1011115)
Supplement: Attachment — Submitted filename: Response to Reviewers.docx [file pcbi.1011115.s009.docx]

**REVIEWER 1**

Line 88: Oral transmission through predation of infected vectors is thought to be the most frequent mechanism of infection among hosts in the sylvatic cycle. Looks like a grammatical error in this sentence.

We have fixed the error; the line now reads: “Oral transmission through predation of infected vectors is thought to be the most frequent mechanism of infection among hosts in the sylvatic cycle”

Line 127-128: What are the potential pathological reasons that make the killed bugs still infectious? Please elaborate with citations.

In Latin America, multiple outbreaks of *T. cruzi* infection in humans have been caused by oral transmission (9,15) and it is recognized that animals can easily get infected by eating infected dead bugs (14). In these outbreaks people got infected by fruits infected with bugs’ feces or by bugs themselves falling into blenders and contaminating fruit juice (12). *T. cruzi* parasites outside of the bug are viable (infectious) for up to 48 hours at room temperature (17) and it is expected that parasites within a recently killed bug would be viable for longer. In our lab we have infected mice with feces from *T. cruzi*-carrying triatomines that were 4 days dead. Studies in Brazil showed that all triatomines exposed to fluralaner treated dogs in the first and second months post-treatment died within 1 day post-exposure (^+^Queiroga et al); similarly, in our study we modeled dogs eating them within hours of dying.

^+^Queiroga TB, Gomez LC, de Sena ER, Dos Santos WV, Ferreira HR, de Araújo-Neto VT, Barbosa-Silva AN, Brito CR, Lima RK, Fagundes-Neto JC, Galvão LM. Insecticidal efficacy of fluralaner (Bravecto) against *Triatoma brasiliensis*, a major vector of *Trypanosoma cruzi* in Brazil. Parasites & Vectors. 2021 12;14(1):1-9. https://doi.org/10.1186/s13071-021-04978-x

In the revised introduction we added new text and references to clarify this point:

“Oral transmission through predation of infected vectors is thought to be the most frequent mechanism of infection among hosts in the sylvatic cycle [9–14] and many people have been infected orally in focalized outbreaks in Latin America [9,15]. The probability of transmission due to oral vector ingestion is about 1000 times greater than vectorial transmission [16,17] and *T. cruzi* parasites in feces outside of the bug are viable (infectious) for up to 48 hours [17]. Dogs, important reservoirs for Chagas disease in the domestic cycle [[18–21]](https://www.zotero.org/google-docs/?WH5eXW), can occasionally get infected through the oral route [[22]](https://www.zotero.org/google-docs/?I7wRDc)”

“However, given that *T. cruzi* can be easily transmitted orally through the ingestion of triatomines [9,13,15,46–49], there is potential for a counterproductive effect: dogs could consume the infected bugs killed by the treatment [27,35,50], increasing infection rates in the dog population.”

9. Noya BA, Díaz-Bello Z, Colmenares C, Ruiz-Guevara R, Mauriello L, Muñoz-Calderón A, et al. Update on oral Chagas disease outbreaks in Venezuela: epidemiological, clinical and diagnostic approaches. Mem Inst Oswaldo Cruz. 2015;110: 377–386. doi:10.1590/0074-02760140285

12. Pereira KS, Schmidt FL, Barbosa RL, Guaraldo AMA, Franco RMB, Dias VL, et al. Transmission of Chagas Disease (American Trypanosomiasis) by Food. Advances in Food and Nutrition Research. Elsevier; 2010. pp. 63–85. doi:10.1016/S1043-4526(10)59003-X

14. Kobylinski K, Rutledge-Connelly R. Blood Feeding Insect Series: American Trypanosomiasis - Chagas Disease: ENY-726/IN650, 7/2006. EDIS. 2006;2006. doi:10.32473/edis-in650-2006

15. Barroso Ferreira RT, Branquinho MR, Cardarelli-Leite P. Transmissão oral da doença de Chagas pelo consumo de açaí: um desafio para a Vigilância Sanitária. Vigilância Sanitária Em Debate. 2014;2: 358/160. doi:10.3395/vd.v2i4.358

17. Passos LAC, Guaraldo AMA, Barbosa RL, Dias VL, Pereira KS, Schmidt FL, et al. Sobrevivência e infectividade do *Trypanosoma cruzi* na polpa de açaí: estudo in vitro e in vivo. Epidemiol E Serviços Saúde. 2012;21: 223–232. doi:10.5123/S1679-49742012000200005

Line 141: typo “.” After 38.81%

We have fixed the error, the line now reads: “... it was 38.81% at approximately 20,000 days… ”

Line 452-453: What are the possible reasons that make dogs consume dead bugs? Are there any behavioral

Dogs exhibit a non-selective eating behavior and there are multiple reports of oral transmission of *T. cruzi* through consumption of infected bugs. We added these references to the revised manuscript.

27. Gürtler RE, Lauricella M, Solarz ND, Bujas MA, Wisnivesky-Colli C. Dynamics of transmission of *Trypanosoma cruzi* in a rural area of Argentina. I--The dog reservoir: an epidemiological profile. Rev Inst Med Trop Sao Paulo. 1986;28: 28–35. doi:10.1590/s0036-46651986000100006

35. Reithinger R, Ceballos L, Stariolo R, Davies CR, Gürtler RE. Chagas disease control: deltamethrin-treated collars reduce Triatoma infestans feeding success on dogs. Trans R Soc Trop Med Hyg. 2005;99: 502–508. doi:10.1016/j.trstmh.2004.11.013

50. Bradley K, Bergman D, Woods J, Crutcher J, Kirchhoff L. Prevalence of American trypanosomiasis (Chagas disease) among dogs in Oklahoma. J Am Vet Med Assoc. 2000;217. doi:10.2460/javma.2000.217.1853

Table 1: What are the units for the parameter values (such as incubation period) in table 1.

Table 1 has been updated - the description column has been revised to “Description (unit)” and the units have been added in parentheses next to the descriptions.

**REVIEWER 2**

The use of insecticide against the dog for killing the vector of *Trypanosoma cruzi* can be considered to trigger two different impacts on the infection risk with *Trypanosoma cruzi*, killing the vector can decrease infection risk, however, the dead vector can be consumed and increase infection risk. The estimation of effectiveness of insecticide requires mathematical modelling due to the nature of this conflicting efficacies of insecticide, and the authors constructed the model and simulated with several scenarios. Although this hypothesis regarding the conflicting efficacies of insecticide is interesting, the evidence is poor. At least, the assessment of the effectiveness of insecticide using observed data of infection status of dog with/without insecticide or epidemiological data of dog is required to show how the authors’ hypothesis is realistic and important to characterize Chagas disease among dog population.

Also, no model validation with real data is conducted. How this simulation study captures the real Chagas disease well is not shown, I think that this paper is not appropriate for publication in PLoS Computational Biology.

We thank the reviewer for their consideration of our paper. In order to conduct the kind of large-scale study that the reviewer (and we and many other Chagas disease researchers) are aiming for, we need to understand the potential for harm, under what conditions we might end up with accidental canine infections, and how dangerous those may be to humans. In the revised version of the paper, we acknowledge the need for the trials the reviewer described, and we clarify that we created this model precisely to provide that framework; our main hope is that our calculations can better guide the kind of field trials that are warranted, especially to ensure that there are proper early stopping conditions in case dogs become infected through ingestion.

Earlier this year, a placebo controlled before-and-after efficacy trial of fluralaner usage on dogs was conducted in 28 households with domestic transmission cycles over ~ 2 years (56). This study demonstrated that site infestation and domicile bug abundance plummeted over the months post-treatment; infected bug abundance also decreased dramatically and remained negligible thereafter (56). This small trial confirms the results of our simulation in regions with a high prevalence of domestic vectors/infestation. This study did not address the possible outcomes of fluralaner treatment in regions of semi-sylvatic bugs and/or low prevalence making the need for modeling these scenarios ever more important prior to concluding that fluralaner treatment would be beneficial in all regions.

56. Gürtler RE, Laiño MA, Alvedro A, Enriquez GF, Macchiaverna NP, Gaspe MS, et al. Treatment of dogs with fluralaner reduced pyrethroid-resistant *Triatoma infestans* abundance, Trypanosoma cruzi infection and human-triatomine contact in the Argentine Chaco. Parasit Vectors. 2022;15: 257. doi:10.1186/s13071-022-05343-2

All this information was added to the revised Discussion:

“Detailed data on the time distribution from bugs feeding on dogs to death would improve estimates for the percentage of bugs that dogs would be able to consume. Our findings warrant further lab experiments and small field trials before launching large xenointoxation-based elimination programs. Before utilizing fluralaner in regions with low disease prevalence and domestic or sylvatic bugs populations, especially in regions where dogs have longer lifespans, lab studies could inform how quickly bugs died within 24-hour post feeding period to determine a reasonable estimate for the percentage of bugs that dogs would be able to consume. Better yet, randomized controlled field trials could be designed to closely follow treated dogs, conduct continuous interim analysis, and include early stopping rules if it turns out that treated dogs are becoming infected at proportions higher than controls.”

“The findings of these simulations are supported by a placebo controlled before-and-after efficacy trial of fluralaner administration to dogs in Chaco Province, Argentina (a region with high prevalence of domestic vectors/household infestation); the authors demonstrated that site infestation and domicile bug abundance plummeted over the months most treatment [56].”

**REVIEWER 3**

Main comments:

1) Rather than stating that authors are modelling effects of treating dogs with fluralaner, I believe that results are ligated to xenointoxication in general. Several compounds besides fluralaner have been assayed for xenointoxication (in ref to Loza et al., 2017) why have the authors narrowed their findings to fluralaner in the title? Indeed the authors state in their abstract that the objective of this work was to: “Examine the potential for increased infection rates of *T. cruzi* in dogs following xenointoxication”. Thus my suggestion is to change the title to: Modeling the impact of treating dogs with xenointoxication to halt *Trypanosoma cruzi* transmission. I would also suggest avoiding to particularly refer to fluralaner when xenointoxication in general would also be the case in the rest of the text.

Thank you for this important suggestion. The new title reads “Modeling the impact of xenointoxication in dogs to halt *Trypanosoma cruzi* transmission”.

In the revised manuscript we also mention that we are using fluralaner as the drug of study as it is the most widely used in xenointoxication experiments, and field teams and programs are considering it for Chagas control and elimination in several countries. The revised text reads:

“... in particular, fluralaner, a relatively new isoxazoline oral insecticide commonly used to prevent tick and flea infestations, proved especially effective in killing bugs when they fed on dogs under laboratory conditions and is being considered for Chagas control programs.”

2) Secondly, authors state that dogs can become infected when eating infected triatomines, even when insects are killed by the xenointoxication (i.e.: “infectious dead bugs” in Fig 1).

-Is there any evidence on the occurrence (and duration) of viability of *T. cruzi* in dead or moribund insects? Can dead triatomines infect dogs that ingest them? Please provide the corresponding references.

Yes, infected dead triatomines are infectious and have caused cases in humans (9,15). The viability of the parasite outside of the insects is up to 48 hours (17), but viability is much longer inside dead bugs; in our lab we have infected mice with *T. cruzi*-carrying triatomines that were 4 days dead. We have added this additional information and references in the revised version. The revised text reads:

“The probability of transmission due to oral vector ingestion is about 1000 times greater than vectorial transmission [16,17] and *T. cruzi* parasites in feces outside of the bug are viable (infectious) for up to 48 hours [17]. Dogs, important reservoirs for Chagas disease in the domestic cycle [[18–21]](https://www.zotero.org/google-docs/?Ip24at), can occasionally get infected through the oral route [[22]](https://www.zotero.org/google-docs/?lIzdyk)”

“However, given that *T. cruzi* can be easily transmitted orally through the ingestion of triatomines [9,13,15,46–49], there is potential for a counterproductive effect: dogs could consume the infected bugs killed by the treatment [27,35,50], increasing infection rates in the dog population.”

9. Noya BA, Díaz-Bello Z, Colmenares C, Ruiz-Guevara R, Mauriello L, Muñoz-Calderón A, et al. Update on oral Chagas disease outbreaks in Venezuela: epidemiological, clinical and diagnostic approaches. Mem Inst Oswaldo Cruz. 2015;110: 377–386. doi:10.1590/0074-02760140285

12. Pereira KS, Schmidt FL, Barbosa RL, Guaraldo AMA, Franco RMB, Dias VL, et al. Transmission of Chagas Disease (American Trypanosomiasis) by Food. Advances in Food and Nutrition Research. Elsevier; 2010. pp. 63–85. doi:10.1016/S1043-4526(10)59003-X

15. Barroso Ferreira RT, Branquinho MR, Cardarelli-Leite P. Transmissão oral da doença de Chagas pelo consumo de açaí: um desafio para a Vigilância Sanitária. Vigilância Sanitária Em Debate. 2014;2: 358/160. doi:10.3395/vd.v2i4.358

17. Passos LAC, Guaraldo AMA, Barbosa RL, Dias VL, Pereira KS, Schmidt FL, et al. Sobrevivência e infectividade do Trypanosoma cruzi na polpa de açaí: estudo in vitro e in vivo. Epidemiol E Serviços Saúde. 2012;21: 223–232. doi:10.5123/S1679-49742012000200005

-Moribund and dead insects are also expected to occur in routine vector control insecticide spraying campaigns, is there any evidence of dogs becoming infected by ingesting insecticide-affected triatomines?

It could be possible that dogs become infected by ingesting dead infected bugs during IRS campaigns. However, this is unlikely due to the time from insecticide application to bug dying, the location of bugs when sprayed, and the location where bugs die. In the case of xenointoxication, moribund or dead bugs would be the closest to dogs increasing their chances of being consumed.

-Also, although this reader is aware of dogs’ habit of eating live triatomines, flies and other flying insects is there any evidence of dogs eating dead or unmoving insects?

Dogs exhibit a non-selective eating behavior and there is a report of dogs eating dead triatomines in an experiment (35). We added this reference to the revised manuscript.

35. Reithinger R, Ceballos L, Stariolo R, Davies CR, Gürtler RE. Chagas disease control: deltamethrin-treated collars reduce Triatoma infestans feeding success on dogs. Trans R Soc Trop Med Hyg. 2005;99: 502–508. doi:10.1016/j.trstmh.2004.11.013

-In addition, a common scenario share by many endemic *T. cruzi* areas are chickens moving/roaming freely along the house ecotopes. Chickens indeed eat insects. Which is the expected effect of chickens subtraction by ingestion of dead insects on the reported results ? Is there any reason for not including this process in the model?

Many other animal species could remove triatomine bugs from the system and the same animals (e.g. chicken) could help maintain or increase the triatomine populations. So we did not intend to create a model for each of these specific scenarios but a more generalizable model, looking specifically at the effects of fluralaner treatment in dogs.

3) In ref to lines 335-337. Have the authors explored the results of the models if instead of incorporating a vector birth rate, they allow the vector population to crash? This could readily be the case after insecticide treatment.

Thank you for your comment - upon your suggestion, we ran the model without the triatomine birthrate forcing the triatomine population towards the carrying capacity equilibrium. As expected, should the triatomine population crashed, with no vectors to transmit *T. cruzi*, the proportion of infected dogs declines.

Instead of including this graph in the revised manuscript we have added the following description of such a scenario:

“We also explored setting the triatomine birthrate to zero, allowing the triatomine population to crash after xenointoxication treatment. As expected, we found that with no vectors to transmit *T. cruzi*, the proportion of infected dogs declines within years."

Minor comments:

Lines 62-63. “a more effective transmission pathway than predation of the dogs by the insects.” Please rephrase, *T. cruzi* is not transmitted by predation.

That sentence was rephrased:

“... may make the insects more available to oral ingestion by dogs; a more effective transmission pathway than stercorarian, the usual route for *T. cruzi* transmission.”

1. Bern C. Chagas’ Disease. N Engl J Med. 2015;373: 456–466. doi:10.1056/NEJMra1410150

Lines 90-91 “the probability of transmission due to oral vector ingestion is about 1000 times greater than vectorial transmission”. Is there any empirical data corroborating this mathematical estimation? If not please state so in the introduction. In ref 9 infected bugs were expected to exhibit an altered behavior due to infection increasing exposure to predations. The opposite is expected to occurred when treating with intoxicated triatomines. How have the authors accounted for this?

The reviewer is correct that those estimates from Kribs-Zaleta were calculated for sylvatic hosts; however, they have been used to model oral transmission in dogs (22). Also, there is no reason to believe that probability of oral transmission would be very different across susceptible species: raccoons have been infected orally to understand oral transmission in carnivores (47) and a murine model has been developed to understand oral infection in humans (48).

We added these new references to the revised manuscript:

“Oral transmission through predation of infected vectors is thought to be the most frequent mechanism of infection among hosts in the sylvatic cycle [9–14] and many people have been infected orally in focalized outbreaks in Latin America [9,15]. The probability of transmission due to oral vector ingestion is about 1000 times greater than vectorial transmission [16,17] and *T. cruzi* parasites in feces outside of the bug are viable (infectious) for up to 48 hours [17]. Dogs, important reservoirs for Chagas disease in the domestic cycle [[18–21]](https://www.zotero.org/google-docs/?cvsSF2), can occasionally get infected through the oral route [[22]](https://www.zotero.org/google-docs/?KDK0Xy)”

“However, given that *T. cruzi* can be easily transmitted orally through the ingestion of triatomines [9,13,15,46–49], there is potential for a counterproductive effect: dogs could consume the infected bugs killed by the treatment [27,35,50], increasing infection rates in the dog population.

22. Coffield DJ, Spagnuolo AM, Shillor M, Mema E, Pell B, Pruzinsky A, et al. A Model for Chagas Disease with Oral and Congenital Transmission. PLOS ONE. 2013;8: e67267. doi:10.1371/journal.pone.0067267

47. Roellig DM, Ellis AE, Yabsley MJ. Oral transmission of *Trypanosoma cruzi* with opposing evidence for the theory of carnivory. J Parasitol. 2009;95: 360–364. doi:10.1645/GE-1740.1

48. Silva-dos-Santos D, Barreto-de-Albuquerque J, Guerra B, Moreira OC, Berbert LR, Ramos MT, et al. Unraveling Chagas disease transmission through the oral route: Gateways to *Trypanosoma cruzi* infection and target tissues. PLoS Negl Trop Dis. 2017;11: e0005507. doi:10.1371/journal.pntd.0005507

Line 93. Not only in urban areas.

The sentence was rephrased, and we added the key role of dogs acting as bridges between both cycles:

“Dogs are key reservoirs in the urban and sylvatic cycles of *T. cruzi* because they are very common within households in Latin America [[6,23]](https://www.zotero.org/google-docs/?VgLqLE), they have longer lifespans compared to other important animal reservoirs such as guinea pigs [[24](https://www.zotero.org/google-docs/?2Pk661), [25]](https://www.zotero.org/google-docs/?vc70Cj), they act as bridges between both cycles [[25]](https://www.zotero.org/google-docs/?cvud3O), and dogs tend to have high infection rates, are very infectious, and have high rates of contact with both vectors and humans [[20]](https://www.zotero.org/google-docs/?TQkeea).”

Lines 96. Please revise extremes, zero seroprevalences have been reported in several countries and also higher prevalences than 60%. I would suggest checking more recent reviews on the topic.

We rewrote that sentence and added references:

“In Latin America, reports on canine seroprevalence in areas where natural infection occurs concentrate between 8-28%, with extremes of 1.5% and 83.3% [26-28].

26. Cardinal MV, Enriquez GF, Macchiaverna NP, Argibay HD, Fernández M del P, Alvedro A, et al. Long-term impact of a ten-year intervention program on human and canine *Trypanosoma cruzi* infection in the Argentine Chaco. PLoS Negl Trop Dis. 2021;15: e0009389. doi:10.1371/journal.pntd.0009389

27. Gürtler RE, Lauricella M, Solarz ND, Bujas MA, Wisnivesky-Colli C. Dynamics of transmission of Trypanosoma cruzi in a rural area of Argentina. I--The dog reservoir: an epidemiological profile. Rev Inst Med Trop Sao Paulo. 1986;28: 28–35. doi:10.1590/s0036-46651986000100006

28. Zeledón R. Epidemiology, Modes of Transmission and Reservoir Hosts of Chagas’ Disease. Ciba Foundation Symposium 20 - Trypanosomiasis and Leishmaniasis (with Special Reference to Chagas’ Disease). John Wiley & Sons, Ltd; 1974. pp. 51–85. doi:10.1002/9780470720035.ch4

Lines 116-117. Please check the reference provided, there is no mention to vector control cost in the cited manuscript.

Thank you for noticing this error. We added updated references and revised the text:

“As the unit cost for indoor residual insecticide treatment in a Latin American house is relatively high (37-40) and can be met with low levels of community participation (41-44), treatment of canine reservoirs with insecticide could prove a useful alternative or complementary strategy to reduce *T. cruzi* infection in people.”

37. Castillo-Riquelme M, Guhl F, Turriago B, Pinto N, Rosas F, Martínez MF, et al. The Costs of Preventing and Treating Chagas Disease in Colombia. PLoS Negl Trop Dis. 2008;2: e336. doi:10.1371/journal.pntd.0000336

38. Nakagawa J, Cordón-Rosales C, Juárez J, Itzep C, Nonami T. Impact of residual spraying on Rhodnius prolixus and Triatoma dimidiata in the department of Zacapa in Guatemala. Mem Inst Oswaldo Cruz. 2003;98: 277–281. doi:10.1590/s0074-02762003000200019

39. Vazquez-Prokopec GM, Spillmann C, Zaidenberg M, Kitron U, Gürtler RE. Cost-Effectiveness of Chagas Disease Vector Control Strategies in Northwestern Argentina. PLoS Negl Trop Dis. 2009;3: e363. doi:10.1371/journal.pntd.0000363

40. Yoshioka K, Nakamura J, Pérez B, Tercero D, Pérez L, Tabaru Y. Effectiveness of Large-Scale Chagas Disease Vector Control Program in Nicaragua by Residual Insecticide Spraying against Triatoma dimidiata. Am J Trop Med Hyg. 2015;93: 1231–1239. doi:10.4269/ajtmh.15-0403

41. Fernández M del P, Gaspe MS, Gürtler RE. Inequalities in the social determinants of health and Chagas disease transmission risk in indigenous and creole households in the Argentine Chaco. Parasit Vectors. 2019;12: 184. doi:10.1186/s13071-019-3444-5

42. Gonçalves R, Logan RAE, Ismail HM, Paine MJI, Bern C, Courtenay O. Indoor residual spraying practices against Triatoma infestans in the Bolivian Chaco: contributing factors to suboptimal insecticide delivery to treated households. Parasit Vectors. 2021;14: 327. doi:10.1186/s13071-021-04831-1

43. Gürtler RE, Cecere MC, Lauricella MA, Petersen RM, Chuit R, Segura EL, et al. Incidence of *Trypanosoma cruzi* infection among children following domestic reinfestation after insecticide spraying in rural northwestern Argentina. Am J Trop Med Hyg. 2005;73: 95–103.

44. Paz-Soldán VA, Bauer KM, Hunter GC, Castillo-Neyra R, Arriola VD, Rivera-Lanas D, et al. To spray or not to spray? Understanding participation in an indoor residual spray campaign in Arequipa, Peru. Glob Public Health. 2018;13: 65–82. doi:10.1080/17441692.2016.1178317

Lines 118-119, “treatment of canine reservoirs with insecticide could prove a cost-effective mechanism to reduce *T. cruzi* infection in people.” No reference has been giving to support this sentence.

I find this statement misleading, it is so vague that what is said can or cannot be true. How many dogs are needed to receive treatment in order to reduce *T. cruzi* infection in people? Which is the cost of the insecticide employed? with which residual effect? How many rounds of treatments are planned? Please revise.

We have revised the text:

“... treatment of canine reservoirs with insecticide could prove a useful alternative or complementary strategy to reduce *T. cruzi* infection in people.”

Table 1. Please check the references provided. Also include the units of the parameters.

Table 1 has been updated - the description column has been revised to “Description (unit)” and the units have been added in parentheses next to the descriptions.

Maximum percentage of vectors eaten by dogs in a day. Please provide a reference to support this estimates. Why have the authors not analyzed the sensitivity of the model to changes in this key parameter?

We thank the reviewer for this important recommendation. We analyzed this parameter with 20%, 50% and 80%. The outputs of this new analysis were added to the revised manuscript.

“We also explored different consumption levels from 20% to 80% (S4-S7).”

“In this simulation, we assumed that dogs consumed 80% of bugs killed with fluralaner treatment; in simulations with this parameter set to 20% and 50%, trends remained the same (S2 Fig). As could be expected, if the dogs consumed a greater number of the bugs, the initial rise in the proportion of infected dogs is greater, followed by a shallower decline in the days posttreatment.”

“In these simulations, we assumed that dogs consumed 80% of bugs killed with fluralaner treatment; in simulations with this parameter set to 20% and 50%, trends remained the same (S3 Fig).”

“When percentages of dead bugs consumed by dogs is lower (at 20% and 50% instead of 80%), the level of infected dogs decline with fluralaner treatment when dogs have a 3-year life span (S4 Fig A-C). When dogs have a 6-year lifespan, infected dog numbers trend up after fluralaner treatment assuming a percentage of 50% as well as 80% but trend down with 20% (S4 Fig D-F).”

“The semi-sylvatic low-prevalence model is sensitive to the proportion of bugs eaten with trends being similar to those of the non-semi-sylvatic cycle low-prevalence model (S5 Fig).”

“In contrast, our findings suggest that in regions with low disease prevalence and domestic or sylvatic bug populations, especially in regions where dogs have longer lifespans, careful attention needs to be given to the potential of unintended consequences of xenointoxication on *T. cruzi* transmission. In these regions, when dogs are able to consume a large percentage of the bugs (more than 50% based on our sensitivity analysis for 6 year life-spans), our models suggest that infection levels in dogs (and in some situations infection in bugs), end up higher than pretreatments levels.”

**S2 Fig.**  **Sensitivity analysis on proportion of consumed bugs by dogs for single fluralaner treatment in a high prevalence region (corresponds to Fig 1 in main text).** (A) corresponds to 20% of bugs being consumed; (B) corresponds to 50% of bugs being consumed; (C) corresponds to 80% of bugs being consumed.

**S3 Fig.**  **Sensitivity analysis on proportion of consumed bugs by dogs for multiple fluralaner treatments in a high prevalence region (corresponds to Fig 2 in main text).** Annual administration of fluralaner for both 4 years (A) and 6 years (B) was simulated, as well as administration every 90 days (veterinary recommendation) for one year (C) and for two years (D).

**S4 Fig.**  **Sensitivity analysis on proportion of consumed bugs by dogs for fluralaner treatments schemes in a low prevalence region (corresponds to Fig 3 in main text).** We explored a range of dog average lifespan from 3 years (A-C) to 6 years (D-F). Treatment scenarios include one time treatment (A, D), annual treatment for 4 years (B, E), and treatment every 90 days for 1 year (C, F).

**S5 Fig.**  **Sensitivity analysis on proportion of consumed bugs by dogs for fluralaner treatments schemes in a low prevalence region with semi sylvatic cycles (corresponds to Fig 4 in main text).** We explored a range of dog average lifespan from 3 years (A-C) to 6 years (D-F). Treatment scenarios include one time treatment (A, D), annual treatment for 4 years (B, E), and treatment every 90 days for 1 year (C, F).

Line 356 and 418-419. I find confusing that it is stated that the “The model considers a single species of host (dogs) and vector (*Triatoma infestan*s) (l.356) ” but in lines 418-419, it is stated “Parameter values for regions with semi-sylvatic bugs were calibrated to approximately values reported in Yucatan, Mexico. There is no *T. infestans* in Mexico. Please explain/revise.

We revised the manuscript:

“The model considers a single species of host (dogs) and a single vector, which represents different species in different scenarios. We do not consider more complex situations with multiple vector species.”

“For simplicity we parameterized the bug population based on data on *T. infestans* for the domestic cycle and data on *T. dimidiata* for the sylvatic cycle [66].

“Parameter values for regions with semi-sylvatic bugs were calibrated to approximately values reported for *T. dimidiata* in Yucatan, Mexico.”

Lines 293-294. “ when dogs consumed a moderate amount of the killed bugs” . I find this expression misleading, 80% of the bugs killed is quite high rather than moderate. Please rewrite this sentence.

We rewrote the sentence to:

“... unfavorable outcomes with fluralaner treatment when dogs consumed 4 out of 5 of the killed bugs”

**EDITOR**

The Authors are expected to address all the criticisms by all Reviewers. In particular, provide more description and assessment of the infectiousness of killed bugs (Reviewer #1 and #3), clarify if the effectiveness of insecticide is well supported and perform some model validation (Reviewer #2), provide more description of whether and how often dogs eating dead insects (Reviewer #3). In additional to the above comments, please address,

1. Discussion “…we assumed that dogs consumed 80% of bugs killed by treatment and that consumption happened immediately upon death. Could the authors assess how realistic are these two assumptions? Is there any direct or indirect evidence to support 80% of killed bugs were consumed?

2. Sensitivity analyses have been carried out for the parameters and was made available in Shiny. However, the main conclusion was based on only one specific set of parameters but there seems to be substantial uncertainty (Table 1). Some of these sensitivity analyses should be presented in the results and also discussed.

3. The main conclusion should take into account the sensitivity analyses, which inform how likely or under what situation there may be potential harm from xenointoxiciation.

We thank the editor and the reviewers for this important recommendations. To respond to the editor’s comment we conducted a sensitivity analysis of the parameter controlling proportion of bugs dogs eat (previously, p=0.8) to supplement the shiny app that displays results for adjusting all the parameters. For completeness, we are submitting the results of this sensitivity analysis as supplemental figures and have added text describing our revised findings. Specifically, we have added the following text:

“We also explored different consumption levels from 20% to 80% (S2-S5).”

“In this simulation, we assumed that dogs consumed 80% of bugs killed with fluralaner treatment; in simulations with this parameter set to 20% and 50%, trends remained the same (S2 Fig). As could be expected, if the dogs consumed a greater number of the bugs, the initial rise in the proportion of infected dogs is greater, followed by a shallower decline in the days posttreatment.”

“In these simulations, we assumed that dogs consumed 80% of bugs killed with fluralaner treatment; in simulations with this parameter set to 20% and 50%, trends remained the same (S3 Fig).”

“When percentages of dead bugs consumed by dogs is lower (at 20% and 50% instead of 80%), the level of infected dogs decline with fluralaner treatment when dogs have a 3 year life span (S4 Fig A-C). When dogs have a 6 year lifespan, infected dog numbers trend up after fluralaner treatment assuming a percentage of 50% as well as 80% but trend down with 20% (S4 Fig D-F).”

“The semi-sylvatic low-prevalence model is sensitive to the proportion of bugs eaten with trends being similar to those of the non-semi-sylvatic cycle low-prevalence model (S5 Fig).”

“In contrast, our findings suggest that in regions with low disease prevalence and domestic or sylvatic bug populations, especially in regions where dogs have longer lifespans, careful attention needs to be given to the potential of unintended consequences of xenointoxication on *T. cruzi* transmission. In these regions, when dogs are able to consume a large percentage of the bugs (more than 50% based on our sensitivity analysis for 6 year life-spans), our models suggest that infection levels in dogs (and in some situations infection in bugs), end up higher than pretreatments levels.”

**S2 Fig.**  **Sensitivity analysis on proportion of consumed bugs by dogs for single fluralaner treatment in a high prevalence region (corresponds to Fig 1 in main text).** (A) corresponds to 20% of bugs being consumed; (B) corresponds to 50% of bugs being consumed; (C) corresponds to 80% of bugs being consumed.

**S3 Fig.**  **Sensitivity analysis on proportion of consumed bugs by dogs for multiple fluralaner treatments in a high prevalence region (corresponds to Fig 2 in main text).** Annual administration of fluralaner for both 4 years (A) and 6 years (B) was simulated, as well as administration every 90 days (veterinary recommendation) for one year (C) and for two years (D).

**S4 Fig.**  **Sensitivity analysis on proportion of consumed bugs by dogs for fluralaner treatments schemes in a low prevalence region (corresponds to Fig 3 in main text).** We explored a range of dog average lifespan from 3 years (A-C) to 6 years (D-F). Treatment scenarios include one time treatment (A, D), annual treatment for 4 years (B, E), and treatment every 90 days for 1 year (C, F).

**S5 Fig.**  **Sensitivity analysis on proportion of consumed bugs by dogs for fluralaner treatments schemes in a low prevalence region with semi sylvatic cycles (corresponds to Fig 4 in main text).** We explored a range of dog average lifespan from 3 years (A-C) to 6 years (D-F). Treatment scenarios include one time treatment (A, D), annual treatment for 4 years (B, E), and treatment every 90 days for 1 year (C, F).
