# Supplementary material for: Modeling the impact of xenointoxication in dogs to halt Trypanosoma cruzi transmission
Source: PLoS Comput Biol. 2023 May 8;19(5):e1011115. doi: 10.1371/journal.pcbi.1011115 (PMC10194993; doi:10.1371/journal.pcbi.1011115)
Supplement: Attachment — Submitted filename: Response to reviewers.docx [file pcbi.1011115.s010.docx]

**REVIEWER #2**:

My comment has been solved partly and as much as possible at the current stage.

Thank you for your thorough review.

**REVIEWER #3**:

My main point regarding this revised version relates to the key assumption that dogs can get infected by eating dead triatomines.

-In reference to the 2nd Response to reviewer 1: Authors state “In our lab we have infected mice with feces from T. cruzi-carrying triatomines that were 4 days dead” Very interesting and relevant information! Have the authors published these results?

No, these results from our lab have not been published; however, we have added a reference of an experimental infection of triatomines that found viable metacyclic trypomastigotes in dead triatomines even after 4 days of the triatomine death.

65. Del S. Guzmán Marín E, Antonio Barrera Pérez M, Eugenia Rodríguez Félix M. Viabilidad del Trypanosoma cruzi en Triatoma dimidiata muertos. Enfermedades Infecc Microbiol Clínica. 1999;19: 113–115.

-In reference to the 4th Response to reviewer 1. In this manuscript the infection of dogs by eating dead triatomines is crucial and is a premise sustaining the whole reasoning and calculations. However, the authors have not provided any evidence that dog can get T. cruzi infection by eating dead triatomines. (Please see my comments on the references provided) Please revise.

-Ref 27 Gürtler et al., 1986. The only mentioning of oral infection in dogs in this manuscript refers to the potential infection dogs may acquire when eating infected rodents.

-Ref 35. Reithinger et al. 2004. The overall disappearance of 12% of exposed bugs to collared and uncollared dogs implies consumption of bugs by the dogs. However, there is no evidence of eating or consumption of dead bugs especially given that the deltametrin-impregnated collars had no effect on triatomine survival.

-Ref 50 Bradley et al., 2000. This manuscript mentions that some puppies may have acquired infection by chasing triatomines because they were reported to chase bugs.

It is thus necessary that the authors clearly warn the reader that it is speculative that dogs may become infected by eating dead triatomines (if they prey on dead triatomines at all).

To our knowledge, there have been no experiments conducted to test the hypothesis that dogs eat triatomine insects because it is commonly accepted they would eat any bug in their surroundings. Veterinary clinics constantly receive dogs that ate scorpions, spiders, stink bugs, bees, among many others. Hunting dog clubs share homemade recipes to treat dogs that eat caterpillars, Asian lady beetles, cockroaches, and crickets. And as veterinarians, we have operated on dogs eating even rocks! However, we understand the reviewer’s point and we added this revised text to warn the readers that in our model consumption of dead dogs is one of our assumptions:

“In our model, we explore scenarios in which we assume that dogs consume dead triatomines and that the dead triatomines ingestion shortly after bug death (within a few days) results in transmission scenarios similar to oral predation.”

We have also added an additional reference of a recently published model of *T. cruzi* in dogs treated with fluralaner that considers a similar assumption.

51. Fiatsonu E, Busselman RE, Hamer GL, Hamer SA, Ndeffo-Mbah ML. Effectiveness of fluralaner treatment regimens for the control of canine Chagas disease: A mathematical modeling study. PLoS Negl Trop Dis. 2023;17: e0011084.

-In reference to my 2nd comment: I believe that authors have provided different references that explain human oral transmission (which is something not questioned at all). The cited references (9,12,15,17) make clear that trypanosomes can survive (and probably multiply) in homemade seasonal fruit juices, however, this is not the kind of oral transmission that could involve dogs (main topic of this manuscript). My point is what evidence exists on the survival of parasite and infectiousness of dead infected triatomines in the household environment. Assuming that parasite survival and infectiousness of dead triatomines are exactly the same in the environment (floor, patio, etc.) than in fruit juices or other food staff is risky. This should be made clear to the reader.

-Regarding the consumption of dead insects: Please refer to my comment above on the interpretation of Reithinger et al., 2004 ‘s results.

-Ref 35. Reithinger et al. 2004. The overall disappearance of 12% of exposed bugs to collared and uncollared dogs implies consumption of bugs by the dogs. However, there is no evidence of eating or consumption of dead bugs especially given that the deltametrin-impregnated collars had no effect on triatomine survival.

Thank you for this important specification, we have clarified this assumption in the text below and also added the reference mentioned in our response to your first comment

“We conducted a simulation study, using an adaptation of the classic Ross-MacDonald malaria model. Oral predation of triatomines is a well-characterized transmission route of T. cruzi [9–17,22]; through simulation we explored the potential effects of transmission of T. cruzi via ingestion by dogs of triatomines killed by fluralaner treatment. Laboratory evidence suggests that metacyclic trypomastigotes are viable in dead triatomines days after triatomine death [66]; additionally, there are some reports of human infection through contamination of fruit juices with dead triatomines [9,11,15,17]. In our model, we explore scenarios in which we assume that dogs consume dead triatomines and that the dead triatomines ingestion shortly after bug death (within a few days) results in transmission scenarios similar to oral predation. ”

-I believe that the results obtained when Triatomine populations crash should be included in the revised version (Figure included in the answers to the reviewers), not only as a sentence and authors should discuss on this topic, for example in the 2nd paragraph of the discussion . Please include if this is a share pattern for all transmission scenarios in the final version of the manuscript.

We assume a triatomine population crash would be in response to a major spray campaign. We do not believe that xenointoxication by itself would cause a population crash. Logistically, if the intervention goal is to eliminate the whole triatomine population, there are more effective and efficient large scale interventions such as indoor residual spraying. Perhaps in the context of insecticide resistance, adding xenointoxication to the intervention would help to extinguish the triatomine population, but that is a whole other topic beyond the scope of our model. We are not aware of any evidence from fluralaner trials that suggest a complete extinction of the triatomine population exists; indeed, to model it, as originally suggested in the first review, we had to set the birth rate of the triatomine population equal to zero, which does not reflect systems in nature.

-It seems that authors are biased on reporting detrimental effects of xenointoxication and conclude “In regions with low prevalence and domestic or sylvatic vectors, there is potential harm”. However the potential effect of diminishing triatomine population and thus decreasing T. cruzi prevalence both in insects and dogs is neglected. Please revise.

Thank you for your concern of possible bias. We are indeed not recommending not using fluralaner for xenointoxication, but instead presenting a possible scenario through modeling in which fluralaner use may have unexpected consequences. We aim to draw attention to the necessity of pilot field trials in order to evaluate the efficacy of this control strategy. To emphasize this point, we have added the following text to the discussion:

“…. in low endemic regions and regions with sylvatic bugs, canine treatment with fluralaner could potentially increase infection prevalence in both dog and bug populations via canine oral consumption of vectors killed by treatment. These simulations, though a simplified version of reality, highlight the need for well-designed studies to investigate the conditions under which fluralaner xenointoxication, a promising One Health intervention, is an effective control strategy against Chagas disease.”

-It would have been helpful that line numbers were included in the revised version with tracked changes.

Our apologies for this oversight, we included them on this new resubmission.

INTRODUCTION, Second paragraph: “The probability of transmission due to oral vector ingestion is ESTIMATED TO BE about 1000 times greater than vectorial transmission. [16,17] and T. cruzi parasites in feces outside of the bug are viable (infectious) for up to 48 hours IN FRUIT JUICES [17]”

We rewrote that paragraph as requested. The revised version reads:

“The probability of transmission due to oral vector ingestion is estimated to be about 1000 times greater than vectorial transmission. [16,17] and *T. cruzi* parasites in feces outside of the bug are viable (infectious) for up to 48 hours (e.g., in fruit juices) [17]”

-4th paragraph in the revised version authors had added “in particular, fluralaner, a relatively new isoxazoline oral insecticide commonly used to prevent tick and flea infestations, proved especially effective in killing bugs when they fed on dogs under laboratory conditions and is being considered for Chagas control programs [33,36]. Ref 33 concludes: “Fluralaner and xenointoxication are eligible for Phase III efficacy trials alone or combined with other methods in the frame of an integrated vector management strategy in areas with or without pyrethroid resistance”. There is no suggestion on the employment for Chagas control program. Please revise.

Thank you for noticing that. We have revised that sentence and references:

“... in particular, fluralaner, a relatively new isoxazoline oral insecticide commonly used to prevent tick and flea infestations, proved especially effective in killing bugs when they fed on dogs under laboratory conditions [33] and is being considered for Chagas control programs [36].”

**EDITOR**

The Authors have addressed most of the computational issues satisfactorily, however there is a critical issue on biological plausibility where the entire analysis was based on. In particular as pointed out by Reviewer #3, evidence that 1) dogs may eat dead triatomines; 2) dead triatomines may be infectious, are crucial for the validity of the analysis. The manuscript can be further assessed if such evidence could be provided and incorporated in the model.

Thank you for your comments. We are glad that the computational issues were satisfactorily addressed. In the revised manuscript we have added clarifications and explanations for the biological plausibility that *T. cruzi* oral transmission could create diverging scenarios for the use of xenointoxication to control Chagas disease and added references to support our statements.
